# Housing and Management of Turkey Flocks in Canada

**DOI:** 10.3390/ani10071159

**Published:** 2020-07-08

**Authors:** Nienke van Staaveren, Emily M. Leishman, Sarah M. Adams, Benjamin J. Wood, Alexandra Harlander-Matauschek, Christine F. Baes

**Affiliations:** 1 Department of Animal Biosciences, Ontario Agricultural College, University of Guelph, Guelph, ON N1G 2W1, Canada; nvanstaa@uoguelph.ca (N.v.S.); eleishma@uoguelph.ca (E.M.L.); sadams05@uoguelph.ca (S.M.A.); aharland@uoguelph.ca (A.H.-M.); 2 School of Veterinary Science, University of Queensland, Gatton, QLD 4343, Australia; b.j.wood@uq.edu.au; 3 Hybrid Turkeys, Kitchener, ON N2K 3S2, Canada; 4 Institute of Genetics, Vetsuisse Faculty, University of Bern, 3001 Bern, Switzerland

**Keywords:** *Meleagris gallopavo*, management, housing, poultry, survey

## Abstract

**Simple Summary:**

Relatively little is known about how farmers house and manage turkey flocks. To address this knowledge gap, a cross-sectional survey on housing and management practices of turkey flocks was conducted among farmers in Canada. Data were collected from 53 hen flocks (64%) and 30 tom flocks (36%), giving a total of 83 turkey flocks across the country. Flock age ranged between 7–14 weeks (interquartile range). The majority of turkey flocks were kept in indoor barns with automated ventilation. Bedding was provided to all flocks, and litter management was mostly focused on avoiding wet litter (e.g., adding dry litter or heat to avoid caking litter). Practices related to feed/water management and environmental control were relatively consistent between farms. More variation was observed between farmers in terms of flock health management and biosecurity practices. These results can benchmark current management practices within the Canadian turkey farming sector and present a foundation for future research.

**Abstract:**

An increased understanding of the turkey sector and how farmers manage flocks can help maintain and improve the health and welfare of turkeys. We conducted a cross-sectional survey among turkey farmers in Canada to gain information regarding general farm characteristics, housing aspects (incl. lighting, ventilation), litter management, feed and water management, flock characteristics, and flock health management. The survey was distributed to 500 farmers through the Turkey Farmers of Canada in April–December 2019. A total of 83 final responses (response rate approx. 20%) were used for a descriptive analysis to determine the frequency of housing and management practices (77 commercial flocks, 6 breeder flocks). Hen flocks (n = 53) had a median age of eight weeks (IQR: 7–12 weeks) and tom flocks (n = 30) had a median age of 12 weeks (IQR: 9–14 weeks). Turkey flocks within Canada are typically kept in indoor barn systems on a concrete floor (87.5%), with bedding (e.g., straw, wood shavings) provided (100%). The majority followed a brood and move growing system (68.8%), and a large proportion of farmers indicated that they raised turkeys under the ‘Raised Without Antibiotics/Antibiotic Free’ or the ‘Responsible Use of Antibiotics’ certification (70.5%). Possible room for improvement could be found in terms of litter management and biosecurity practices, however, further research is needed to make clear recommendations.

## 1. Introduction

The turkey production sector is an important source of food in many parts of the world [1]. North and Central America traditionally play a significant role in turkey production [2], and Canada has been one of the top ten producing countries in recent years [3]. Turkey production in Canada is regulated under a supply management system that manages the production, pricing, and import of turkey products to maintain stable prices and prevent surpluses or shortages [4]. The per capita consumption of turkey meat in Canada has remained relatively stable, at 4.2 kg since 2015 [5]. In 2018, the consumption of turkey meat ranked only fourth after chicken (34.6 kg), beef (18.1 kg), and pork (16.5 kg) [5]. The Canadian turkey sector is an integrated system and has a total farm gate value of 392 million CAD. There are over 500 commercial turkey farmers represented through the Turkey Farmers of Canada, which together produce approx. 168 million kg of turkey meat annually [6].

Although turkey production is an important industry, both worldwide and in Canada, there is a lack of general knowledge about how turkeys are raised. Over half of consumers surveyed in the US indicated that they are concerned about the welfare of turkeys; however, they reported that they had little to no knowledge about turkey production [7]. Consumers often consider ensuring animal health and welfare to be one of the main responsibilities of farmers [8]. On the other hand, farmers sometimes dismiss consumers’ concerns regarding animal welfare, claiming they stem from the ignorance of animal production systems [9], which makes them concerned about stigmatization [10]. With increasing public interest in general food production practices, there is a need for transparency and continuous improvement [11]. For example, recent studies have shown that farm tours changed visitors’ perceptions of poultry [12] and dairy farms [13] to some extent. Increased understanding of the turkey sector and how farmers address the health and welfare of turkeys can help nurture the relationship between farmers and consumers, ultimately ensuring farmers maintain their societal license to produce. 

While turkey welfare is receiving increasing attention [14,15], there is limited scientific knowledge on the housing and management of flocks, and much of the research is dated in turkeys [16]. The Canadian Code of Practice for the care and handling of hatching eggs, breeders, chickens, and turkeys serves to provide national animal care requirements and recommended practices [17]. Furthermore, the Turkey Farmers of Canada have also implemented the auditable Flock Care Program^©^ to complement and augment the Code of Practice [18]. However, the scientific committee on the Canadian Code of Practice identified several priority issues relevant for turkey production, such as feed restriction in turkey breeders, feather pecking and cannibalism, air and litter quality, stocking density, lameness, lighting regimens, methods of on-farm euthanasia, and physical alterations [16]. Before we can begin to address these issues, it is crucial to understand the current housing and management practices used on turkey farms. This study aimed to describe and benchmark the different housing and management practices being used on turkey farms across Canada. 

## 2. Materials and Methods 

An extensive questionnaire was designed to understand housing and management practices on turkey farms as part of a larger cross-sectional study aiming to identify risk factors for pecking-related injuries and footpad dermatitis. The questionnaire covered a range of topics, including general farm information, housing aspects (incl. lighting, ventilation), litter management, feed and water management, flock characteristics and health, and farmer perceptions of issues within the turkey sector. The questionnaire was adapted from a similar study conducted in the Canadian laying hen sector [19], with the expertise of the research team and other turkey industry professionals. The self-administered questionnaire mostly included (semi-) closed questions, and was available in both English and French. Farmers were asked to fill in the questionnaire for one of their current flocks at its current age, unless otherwise indicated. This study was approved by the University of Guelph Research Ethics Board (REB19-02-015).

All ~500 registered turkey farmers were invited to participate in the study through the Turkey Farmers of Canada in April 2019. Farmers received a package which included a cover letter, questionnaire, and return envelope, that was given a unique code to ensure confidential and anonymous data collection. A 10 CAD gift card was included as incentive to encourage participation. Additionally, farmers were provided the opportunity to fill in the questionnaire online via Qualtrics (Qualtrics, Provo, UT, USA). Reminders were sent out through the Turkey Farmers of Canada until the end of data collection in December 2019. Additionally, the study was promoted at various industry meetings during this time. 

Data of the self-administered survey were entered into Microsoft Office Excel (Professional Plus 2016) using manual double entry and checked for entry errors. Invalid responses were considered missing values. Not all farmers answered all questions, and thus, the final sample size per question varied. The frequency of the different management practices used in the different housing systems were calculated using descriptive statistics in SAS v9.3 (SAS Inst. Inc., Cary, NC, USA). Results are presented as the number and percentage of flocks for categorical variables and median (interquartile range (IQR)) for continuous variables. Exploratory Spearman rank correlations were calculated between housing and management practices and biological measurements (e.g., bird age, body weight) to determine their relationships.

## 3. Results

A total of 500 questionnaires were sent out to turkey farmers across the country. Ninety-nine questionnaires were returned (approx. 20% response rate), including 86 (17%) completed questionnaires and 15 (3%) incomplete questionnaires by respondents, indicating they no longer produce turkeys. Three questionnaires were excluded due to the incorrect interpretation of the instructions leaving a final 83 questionnaires used for analysis.

### 3.1. Demographics

The majority of farmers were male (86.6%) and 62.7% of respondents were 45 years of age or older. Most farmers had more than ten years’ experience in turkey production and represented all major turkey production provinces in Canada (Table 1).

### 3.2. Turkey Production, Housing, and Management

#### 3.2.1. General Housing and Production

Turkeys are kept in indoor floor barns, with most farms consisting of one to three barns (65.9%), though 15.9% of respondents had more than five barns on their farm. Over 81% of surveyed barns were constructed, or had a major renovation done more than five years previously (Table 2). Floors were mostly made of concrete or wood (typically when more than one storey was present), with a relatively small proportion of barns utilizing dirt as a floor. The most common types of production were heavy tom production, heavy hen production, light hen production, and broiler turkey production. However, nearly 44% of respondents indicated that they did not specialize in one type of production, but rather combined different types of production on their farm. Farmers produced a median of 30,000 turkeys per year (n = 80, IQR: 13,300–75,000). The median flock size was 6715 birds at placement (n = 82, IQR: 3000–11,200). Stocking density was estimated to have an IQR between 17.2–37.6 kg/m^2^, with a median of 26.3 kg/m^2^ (n = 74), depending on age (n = 73, r = 0.69, *p* < 0.001) and body weight (n = 73, r = 0.68, *p* < 0.001) of the surveyed flocks in the cross-sectional study. 

#### 3.2.2. Litter Management and Enrichment

Straw and wood shavings were the most common bedding substrates (Table 3), either together or separately, and two farmers used other types of substrates (sunflower shells and peat moss). Only a few farmers attempted to specify the type of wood shavings they used, with most references being made to kiln-dried wood, soft wood, pine and spruce. Litter depth was reported as a median 10 cm (n = 81, IQR: 7.6–11.4 cm). The self-reported impression of the litter condition indicated that the litter was not overly dry or wet within most barns. Litter condition was mostly assessed (n = 82) through visual inspection (84.2%), though some farmers also indicated that they perform checks with their heel (47.6%), hand (23.2%), or rely on the smell of the litter (25.6%). Over 54% of farmers indicated that they used more than one method to maintain litter quality, while the remaining farmers employed one method. The most common methods used to maintain litter quality were to add dry bedding over wet areas as needed, or add heat to avoid the caking of wet litter. Most farmers added extra bedding during the production of the flock, typically adding the bedding around the water lines. Additionally, about 16% of farmers used feed/water additives to reduce the wetness of the litter (e.g., Betaine ^®^). A relatively small proportion of farmers had reared previous flocks on the litter currently in the barn; specifically, one flock (3.7%), two flocks (7.4%), or three flocks (2.5%) had been previously reared on the litter (n = 81). 

Additional enrichment (n = 69 responses) was provided to nearly one quarter of the turkey flocks (23.2%). This was usually in the form of bales of hay/straw, either on the ground (14.7%) or in hanging nets (1.5%), in the form of other hanging objects (8.8%) or objects on the floor (2.9%), and a few indicated that they provided perches (2.9%). It should be noted that bedding itself was not considered as enrichment but as a housing feature.

#### 3.2.3. Feed and Water Management

Approx. 40.5 birds were estimated per feed pan (n = 70, IQR: 33–52 birds), and 16.3 birds (n = 51, IQR: 12.0–25.3) birds per closed watering system (i.e., per nipple), and 80.0 (n = 22, IQR: 71.0–100.0) birds per open watering system (i.e., per bell drinker). Feed was provided as pellets in the majority of flocks and was purchased from external suppliers (Table 4). The number of times the diet was changed increased as birds got older (n = 78, r = 0.44, *p* < 0.001), with the majority of flocks having undergone 3–6 diet changes at time of the study. A little over one-third of the flocks received feed that included animal by-products, while nearly half of the flocks received feed supplements, which were often insoluble grit and vitamins/minerals.

Most farms used a closed watering system (e.g., nipple-type drinkers), while others used an open watering system (e.g., bell-type drinkers, troughs). Most of the farmers sourced the water from a deep well (71.1%) or town water (16.9%). Nearly 55% of farmers provided supplements such as acids or vitamins/minerals through the water supply. The flushing, cleaning, or sanitizing of water lines occurred before nearly all flocks were placed in the barn. The majority of farmers added water sanitizing products, such as chlorine (57.3%), chlorine dioxide (8.5%), or hydrogen peroxide (18.3%). Farmers continued to inspect the water quality (95.1%) through laboratory checks (e.g., CFUs, chemical content, pH; 69.5%), the assessment of odor (50.0%), color (45.1%), or cloudiness (43.9%) of the water and the presence of rust (30.5%). 

#### 3.2.4. Environmental Control

The most common light source was LED in the surveyed turkey flocks (Table 5). Light intensity ranged from less than 10 to 20 lux on most farms (65.6%), but 22.9% of farmers were unable to answer this question. One-third of farmers (out of 81 farmers) indicated that they had reduced light intensity to correct for abnormal or aggressive behaviors within the flock. Most farmers provided birds with a dark period, at a median of 6.0 h (n = 67, IQR: 4.0–7.0 h) depending on age (n = 65, r = 0.34, *p* < 0.01). Nearly 20% of the farmers used an intermittent lighting program for the flock at their current age, with most providing two separate dark periods within 24 h. The majority of farms had a fully automated controlled environment in which they housed the birds, while some had open-sided barns with (mostly white) curtains. Cross flow ventilation was the most common ventilation method employed, with the majority having their inlets and exhaust on the side walls (88.6% and 76.3%, respectively), or through the attic (24.1%) or chimney (27.6%), respectively (out of 79 responses). The minimum ventilation rate was only provided by 31 farmers and had an IQR of 0.006–0.109 m^3^/min per bird (median: 0.02 m^3^/min per bird). Median temperature and humidity were reported at 20.0 °C (n = 75, IQR: 18.3–21.1 °C) and 55.0% relative humidity (RH; n = 60, IQR: 50.0—60.0%RH), respectively.

### 3.3. Flock Characteristics and Management

#### 3.3.1. Flock Characteristics

Hen flocks were more heavily represented in the surveyed flocks (Table 6). It should be noted that approx. six flocks were breeder hen flocks. Hen flocks had a median age of eight weeks (n = 51, IQR: 7–12 weeks), and tom flocks 12 weeks (n = 30, IQR: 9–14 weeks), at the time of the study (placed between October 2018 and December 2019). Birds within hen flocks weighed 4.8 kg (n = 48, IQR: 3.5–7.4 kg), whereas birds within tom flocks weighed 9.5 kg (n = 29, IQR: 7.0–12.0 kg). Hens were reared to a median target weight of 7.5 kg (n = 53, IQR: 6.2–9.0 kg) and toms to a median target weight of 16.0 kg (n = 29, IQR: 14.5–16.5 kg). Physical alterations were performed on day-old poults in the hatchery. Nearly all flocks were beak trimmed in the hatchery (Table 6), with 10.1% of farmers indicating that they were dissatisfied with the quality of the trim (e.g., too short). Additional physical alterations included toe treatment (76.5% of hen flocks, 21.4% of tom flocks), dew claw removal (29.4% of hen flocks, 7.1% of tom flocks), and snood removal (17.7% of hen flocks, 46.4% of tom flocks). The majority of farms used a specialized brooding barn (‘brood-and-move’), while the remainder of farmers had the brooding and grow-out phase in the same barn (‘brood-to-finish’). Upon arrival from the hatchery, 11.0% of farmers observed variation in the poults’ body size. In flocks where birds had been moved to a grow-out barn, the most commonly observed issues after moving included variation in body size, as well as leg deformities. The predominant strain of turkeys used on the surveyed farms was the Hybrid Converter, followed by Aviagen Nicholas Select, though some farmers indicated that they used multiple strains (7 farmers). 

#### 3.3.2. Flock Health

Most farmers used a variety of methods, including cleaning and disinfection, to prepare the barn before the birds’ arrival (Table 7). Similarly, a range of biosecurity practices was reported, with the most common being vermin control and the use of boot washing/boot dips, while showering in/out was least common. Farmers reported other poultry species (43.4%) and other farm species (42.2%) present on the farm location. Over 70% of farmers had multiple age classes of turkeys on their farms. 

Some flocks were reared to meet the requirements of the ‘Raised Without Antibiotics’/‘Antibiotic Free’ certification, or the ‘Responsible Use of Antibiotics’ certification, with one farmer indicating organic rearing (Table 8). The remaining flocks were reared conventionally. The majority of flocks were vaccinated, with vaccines against, e.g., coccidiosis, hemorrhagic enteritis, Newcastle disease, and colibacillosis. The majority of farmers indicated that they had an existing relationship with their veterinarian and medication was provided to nearly 40% of the flocks that mostly targeted the control of coccidiosis and enteritis. Diseases were observed in nearly one-third of the flocks, with the most reported diseases including colibacillosis (15.5%), coccidiosis (5.6%), hemorrhagic enteritis (5.6%), necrotic enteritis (4.2%), cellulitis (2.8%), salmonella (1.4%), turkey rhinotracheitis (1.4%) and blackhead disease (1.4%). Additionally, 11.1% of 81 farmers indicated that they had noticed birds with poor vision, blindness, or cataracts in their flock. Specialized hospital or recuperation pens were not very common in the surveyed flocks. The cumulative mortality rate at the time of the survey was 2.8% (n = 49, IQR: 1.8–4.6%) in hen flocks, and 4.5% (n = 27, IQR: 2.8–6.3%) in tom flocks. Cumulative mortality was positively correlated with the age of the flock (n = 76, r = 0.55, *p* < 0.001). The majority of farmers indicated that personnel were trained to perform on-farm euthanasia. Personnel inspected the flocks typically 2–3x per day (85.6%) by 1–2 workers (91.5%), with most inspections lasting between 15–30 min. 

## 4. Discussion

### 4.1. Housing Design and Management

This study provides results on a cross-sectional survey of 83 commercial turkey farmers, giving detailed information on housing and management practices in Canada. The response rate was approx. 20%, which is higher than a previous survey conducted in Canada on turkey euthanasia methods [20]. 

Turkey flocks in Canada are typically kept in indoor floor systems with bedding (e.g., straw, wood shavings) on a concrete/wooden floor. A small number of farmers reared turkeys on a dirt floor, which has been reported before for turkey or broiler production, in the US, South America and Europe [21,22,23,24]. The Code of Practice makes no recommendation regarding floor type, however it does place emphasis on bedding and litter management [17]. All farmers in the study provided some kind of bedding substrate, with straw and wood-shaving being the most commonly used. When details on the type of wood shaving used were requested, most farmers were unable to provide this information. However, more important than the type of bedding, good litter management is needed to maintain litter quality and keep litter loose, dry and friable [23,25]. Litter quality is impacted by environmental factors, such as ventilation, diet, and bedding material, and litter that is too dry or too wet should be avoided [16]. Martrenchar et al. [23] cautioned that adding too much bedding could potentially increase the risk of footpad dermatitis, as it would be more difficult to keep dry. Farmers in the current study estimated their litter depth to be around 10 cm, but no Canadian recommendations for litter depth are available. Farmers mostly reported good litter quality (low moisture content and loose), although some felt that the litter was very dry or damp/tacky. It should be acknowledged that this is a self-reported and subjective assessment of litter quality that may not necessarily reflect the actual litter quality. More comprehensive measures of litter moisture, pH, and nitrogen content could have provided more accurate insights into the litter quality, but were not feasible in the current study. Different techniques were employed by farmers to maintain litter quality, with the main ones being methods to deal with wet litter (e.g., adding dry bedding, heating, tilling, moving water lines, and adjusting ventilation). The greater emphasis on managing wet litter suggests that wet litter was more of an issue for farmers than too dry litter at the time of the survey, which covered a range of flock ages and seasons. The large proportion of farmers who use multiple methods to check and maintain litter quality emphasizes the importance farmers place on good litter management in turkey production. Finally, while the Code of Practice requires fresh bedding for poults at placement, this is not necessarily a requirement for the grow-out barns [17]. This explains why approx. 15% of farmers used the same litter for consecutive flocks in the current study (in the grow-out barns). Though not specifically asked, it is also likely that a fresh layer of bedding was applied before flock placement in the grow-out barns. The reuse of litter is a practice used in several countries, though not common in Europe [26,27]. Possibly the farmers in the current study used a similar approach as observed in 85 Australian turkey farms, where 82% added fresh bedding for each batch, but only 27% removed old litter/manure [27]. Interestingly, depending on the type of litter, the reuse of litter over consecutive flocks has been shown to both increase or reduce the risk of different types of condemnations in broilers [28]. All in all, the reuse of litter is not well understood, and further research on its potential benefits or drawbacks, in terms of the productivity, health, and welfare of turkeys is required. The reported bird stocking densities were generally well in line with the recommendations for certain weight groups in the Canadian Code of Practice [17], however, it should be acknowledge that these stocking densities were based on estimated values for average body weight, number of birds, and space available, and as such, should be treated as rough estimates. 

Nearly one quarter of farmers said they provided environmental enrichment to their flocks, typically in the form of hay/straw bales or nets. Environmental enrichment is a modification of the environment that should improve the biological functioning of animals [29]. The provision of hay/straw is considered biologically relevant, as it is a form of substrate and is thought to help improve animal welfare by reducing injurious pecking [14]. However, very little scientific research is available on enrichment for turkeys, and the research that is available has conflicting results [30,31,32].

The majority of farmers provided feed in a pelleted form from an external supplier, while only a few farmers indicated that they home-milled mashed feed. The number of feed changes (i.e., feed phasing) increased as birds got older, with 3–6 changes being common. It is recommended that feed changes are introduced gradually [17]. Pelleted feed is suggested to improve feed conversion compared to mashed feed [33] or crumble [34]. Feed form, however, may also impact bird behaviour, with pellets typically thought to reduce time spent feeding [35]. As this study was aimed at commercial turkey farmers, we did not ask any questions regarding the practice of feed restriction which occurs in breeder populations [16,35] (only a small proportion of breeder flocks participated in the survey). The majority of farmers used a closed watering system as opposed to open water drinkers. Closed watering systems limit bacterial growth and spillage [17,36], thereby impacting litter quality. Interestingly, it was suggested that birds might prefer open watering systems (e.g., bell drinkers) from a behavioral point of view, as it allows a more natural scooping motion. Research in broilers showed no preferences for different drinker types, and indicated instead that the height of the drinkers was more critical [36]. However, limited research has been done in turkeys on their preferred drinking behaviour. The height of bell drinkers can be individually adapted and so might help farmers manage flocks that are not very uniform in size. Variation in body size is an issue, according to the surveyed turkey farmers, and this could potentially be linked to the problem of mortality seen due to dehydration [37]. Improving flock uniformity or designing closed drinker systems that can be adapted to different heights might provide a way to counter this potential issue. Farmers were asked to estimate the number of birds per feeder and drinker system; however, these results should be interpreted with caution, as they are likely not very accurate, given the relatively large interquartile range. Nearly all farmers flushed, cleaned, or sanitized the water lines and performed regular inspections of the water quality, as recommended in the Code of Practice [17].

The most common type of lighting provided was LED, with reported light intensities ranging between less than 10 to 30 lux in most flocks (with nearly 23% of farmers unable to provide an estimate). While light intensity can be reduced to correct for unwanted behaviors, such as injurious pecking, this should only be temporary [17]. One-third of farmers indicated that they had adjusted light intensity in the barn for this reason, but it is unclear whether this was a temporary measure. Nearly all farmers provided birds with a dark period, which averaged at about 6 h of darkness. The Code of Practice requires a minimum of 4 h of darkness per day [17], as continuous light can have negative impacts on bird development and welfare [14]. Many of the recommendations regarding lighting regimes come from broiler production, and further research on lighting in turkeys is needed [16]. 

Open-sided barns with curtains were used to house approx. 37% of the flocks. Approx. 32% of turkey farms in the US were estimated to use tunnel ventilation in the grow-out period [38], while the proportion of farmers in the current study that used tunnel flow ventilation was much lower (~18%). The majority used cross flow ventilation (~70%); however, not all farmers were able to indicate the type of ventilation being used. The average temperature and relative humidity reported by farmers was within the recommended range in the Canadian Code of Practice for the different age ranges of the flocks [17]. However, more variability was observed in the reported relative humidity, and approx. 28% of respondents did not provide an answer to this question. This could suggest that either not all staff regularly measure relative humidity, or that they are unsure of where this information can be found in the barn, or, finally, that the measurement is simply not available within the system. Similarly, only a few farmers (~38%) were able to provide the ventilation rate when using mechanical ventilation, and a large range was reported, thereby putting the accuracy of the responses into question. However, the average values for temperature, relative humidity and ventilation were within the range of values reported by Reynolds et al. [21], who also reported large differences between hen, tom, or brooder barns depending on the season (summer/winter). This could also explain the large variation seen in the current cross-sectional study, for which data were collected across different flock sexes, ages and seasons. Furthermore, Canada’s provinces can differ widely when it comes to weather [39], and these differences will impact management practices, such as, e.g., ventilation, barn climate, and litter management. 

### 4.2. Flock Characteristics and Management

To the authors’ knowledge, this study provides the first indication on the proportion of flocks that are raised conventionally or under different certification programs, such as ‘Raised Without Antibiotics/Antibiotic Free’ or ‘Responsible Use of Antibiotics’. Interestingly, the data represented more or less equal numbers of each flock certification program. This could represent the increasing interest of the public for programs that lower the antibiotic usage in animal production [40,41]. Alternatively, it is possible that the farmers pursuing these certifications were more inclined to participate in research. Farmers and veterinarians in the US indicated that the switch to these programs was mostly market-driven, and expressed concerns that it could negatively impact animal health and welfare [40]. Karavolias et al. [41] found that broilers raised without antibiotics were more likely to have more severe eye burns, footpad dermatitis, or airsacculitis compared to broilers that were given antibiotics. No literature is available on how different antibiotic programs influence productivity, health, and welfare of turkeys, underlining the lack of knowledge in this area.

The main turkey breeds in the flocks studied were Hybrid Converter and Aviagen Nicholas Select, with a larger proportion being the Hybrid Converter strain. Due to the cross-sectional design of the study, a wide range of flock ages (in part due to the small number of breeder flocks within the responses) were included, with flocks being on average 10–11 weeks old. The age of the flock also depended on the type of production, sex, and final target weight. Our data mostly represented hen flocks, while approx. 36% of farmers provided information for tom flocks. This likely influenced the management practices, as well as the potential welfare-related issues seen in these flocks [42]. For example, while both sexes were beak trimmed, toe treatment was more common in hen flocks and snood removal in tom flocks. These physical alterations at the hatchery are intended to avoid injuries in birds, and the need for these practices should be evaluated regularly [17]. Toe treatment is associated with changes in behaviour and pain in hens and toms [43,44]. The procedure is performed to avoid scratches on the carcass, particularly in hens [16,44], and used for the whole bird market, while, in toms, it is not effective, due to their heavier weight [45]. However, processors can also require both tom and hen flocks to have toe treatment and dew claw removal performed, and dew claw removal can also occur in breeder hen flocks to avoid injuries in automated nest systems. This could explain the occurrence of these procedures in surveyed flocks. Fournier et al. [45] also suggested that toe treatment might still reduce scratching behaviour at younger ages in toms, which could have implications for bird welfare. Finally, it cannot be ruled out that some farmers might have grouped dew claw removal and toe treatment together. The removal of the snood is a typical treatment in tom flocks to reduce the risk of injury [17,31,46], and has been additionally associated with a lower risk of mortality at 7 and 14 days of age [46]. However, the scientific committee of the Canadian Code of Practice for turkeys has cautioned that research on the effects of physical alterations is limited or dated, and may not reflect current genetics or production practices, thus, more recent studies are needed in this area [16].

A survey of turkey veterinarians in the United States indicated that single-age brooding was increasingly implemented from 35% in 2010 to 53% in 2018, to help manage disease on farms [38]. In Australia, 85% of turkey farmers indicated that they had single-age classes on their farm, likely due to the high level of integrated ownership within the sector [27]. In contrast, Canadian turkey production is less integrated, and only 30% of farmers in the current survey indicated that they had one age class on their farm. However, where multiple age classes were present, most birds were inspected age-wise (i.e., youngest to oldest), as recommended [17]. Farmers typically performed 2–3 inspections per day, which is in line with the requirement of at least two inspections per day in the Canadian Code of Practice [17]. Additionally, the majority of farmers reported using several cleaning and disinfecting methods to prepare barns for bird arrival, as well as using several biosecurity practices, as similarly observed in a small sample of Ontario poultry farms [47]. However, a large proportion of farmers indicated the presence of other poultry species present on their farm, in contrast to only 2 out of 81 turkey farms in Australia [27]. East [27] suggested that, with less integrated ownership, there is more variation in the use of biosecurity practices. The presence of other poultry species could form a biosecurity risk [48]. It should also be noted that a small proportion of flocks included in the survey were mature birds (i.e., breeder flocks), which would have influenced management practices, particularly in regard to stricter biosecurity practices.

In general, nearly all farmers indicated that they had an existing relationship with a veterinarian, who typically came to the farm only as needed. Nearly 30% of the farmers reported disease within the flock at some point in time, which approximates the 40% of farmers that considered disease an issue on their farm [37]. However, it should be noted that this is a self-administered questionnaire, and it is not clear whether disease or issues indicated by farmers were supported by veterinary diagnosis, slaughterhouse reports, or were self-diagnosed by the farmer. The reported vaccinations and medications by the farmers aimed to target the most commonly mentioned diseases, e.g., colibacillosis, coccidiosis, and hemorrhagic or necrotic enteritis. These diseases were also frequently identified by veterinarians in Canada and the US as important issues for the turkey production sector [38,49]. Since 40% of farmers did not record culling and mortality separately, the cumulative mortality rate presented in this study included both culled birds and mortalities. The Code of Practice requires that culls and mortalities are recorded daily, and states that the recording of culls and the reason for them can help identify areas for improvement [17]. All in all, the median cumulative mortality rate was approx. 3.6% which, albeit difficult to compare across flock sexes and ages, is in agreement with other studies [50]. A little over 10% of farmers indicated that staff had not been certified by a veterinarian/professional to perform euthanasia. This lack of on-farm training can be a risk, as the proper training of personnel is necessary to properly and confidently execute on-farm euthanasia in a timely manner [16,17]. 

Despite the reasonable response rate, it must be acknowledged that this study reflects a subset of turkey farmers in Canada, and the results should not be generalized to the whole sector. However, the study population did represent the turkey production sector in Canada in terms of the type of production and the province of the farm [5]. As previously mentioned, this is a self-administered and cross-sectional study which could have influenced responses through subjective responses and the variation in age and sex of the flocks. Housing and management conditions inform important aspects of turkey health and welfare. The results of the current study can provide information about Canadian practices in regards to several of the research priorities identified by the scientific committee of the Code of Practice for the care and handling of turkeys [16]. In particular, it sheds light on practices that are relevant to issues such as feather pecking and cannibalism, air and litter quality, stocking density, lameness, lighting regimens, methods of on-farm euthanasia, and physical alterations in turkeys [16]. Similarly, US veterinarians found that welfare and management ranked as the most important priorities for the turkey sector after food safety and disease [38]. Furthermore, a small sample of predominantly European turkey farmers and veterinarians indicated that they believed ‘environment’ and ‘feeding’ was important for turkey welfare [51], which is also linked to management. However, the authors cautioned that the limited response of turkey stakeholders could reflect that they were not overly willing to share ideas regarding turkey welfare, possibly due to the emphasis on production in regards to turkeys compared to some of the other species (i.e., small ruminants) included in their study [51]. The reasonable response rate in the current study suggests that turkey farmers in Canada are, in fact, willing to share their ideas and housing and management practices in efforts to inform research to reduce pecking injuries or footpad dermatitis. This can help improve consumer knowledge and the relationship between farmers and consumers [7,11].

## 5. Conclusions

This study provides an overview of the current housing and management practices used within turkey flocks in Canada. The results represent different flock ages and sexes across all provinces, which should be kept in mind when interpreting the findings. The majority of turkey flocks are kept in indoor barns. Bedding is provided to all flocks, and litter management is mostly focused on avoiding issues with wet litter (e.g., adding dry bedding or heat). Practices related to feed and water management and environmental control were relatively consistent between farms. More variation was observed between farmers in terms of flock health management and biosecurity practices. These results can provide a foundation of knowledge on turkey husbandry practices in Canada. Understanding the current conditions can aid in developing areas for future research (e.g., physical alterations, lighting, environmental enrichment, reuse of litter, drinking behaviour, antibiotic programs), and benchmarking husbandry practices within the Canadian turkey farming sector.

## Figures and Tables

**Table 1:** A description of general farm characteristics for 83 turkey flocks placed between October 2018 and December 2019 in Canada. The total number of responses is given for each individual question.

	Responses	
	n	%
*Gender (n = 82)*		
Female	11	13.4
Male	71	86.6
*Age (n = 83)*		
18–24 years	3	3.6
25–34 years	9	10.8
35–44 years	19	22.9
45–54 years	17	20.5
55–64 years	25	30.1
65 years and above	10	12.1
*Years of experience (n = 83)*		
Less than 1 year	3	3.6
1–4 years	2	2.4
5–10 years	17	20.5
More than 10 years	61	73.5
*Province (n = 83)*		
Nova Scotia	3	3.6
New Brunswick	1	1.2
Quebec	13	15.7
Ontario	32	38.6
Manitoba	14	16.9
Saskatchewan	3	3.6
Alberta	7	8.4
British Columbia	10	12.1

**Table 2:** A description of general farm characteristics for 83 turkey flocks placed between October 2018 and December 2019 in Canada. The total number of responses is given for each individual question.

	Responses	
	n	%
*Number of barns (n = 82)*		
1 barn	17	20.7
2 barns	21	25.6
3 barns	16	19.5
4 barns	9	11.0
5 barns	6	7.3
More than 5 barns	13	15.9
*Age of system (n = 82)*		
Less than 1 year old	1	1.2
1–4 years old	14	17.1
5–10 years old	26	31.7
More than 10 years old	41	50.0
*More than 1 storey (n = 83)*		
Yes	14	16.9
No	69	83.1
*Flooring (n = 80)*		
Dirt	10	12.5
Concrete	56	70.0
Combination of concrete, wood, dirt	14	17.5
*Type of production ^1^ (n = 83)*		
Broilers (6.2 kg and under)	26	31.3
Light hens (up to 7.5 kg)	29	34.9
Heavy hens (7.5 to 10.0 kg)	38	45.8
Light toms (10.0 kg and under)	4	4.8
Heavy toms (more than 10 kg)	38	45.8
Mature birds	4	4.8

^1^ Farmers could select multiple options.

**Table 3:** A description of litter management aspects for 83 turkey flocks placed between October 2018 and December 2019 in Canada. The total number of responses is given for each individual question.

	Responses	
	n	%
*Type of bedding (n = 83)*		
Sand	0	0.0
Straw	34	41.0
Wood shavings	39	47.0
Combination of the above	8	9.6
Other	2	2.4
*Litter condition (n = 82)*		
Litter very dry and dusty	14	17.1
Low moisture content, litter loose in all the house	53	64.6
Litter damp or tacky	15	18.3
Majority of litter is wet, sticky, or greasy	0	0.0
Soggy, squelchy, or very wet	0	0.0
*Litter quality maintenance (n = 81)*		
Remove wet/caked litter	6	7.4
Add dry bedding	67	82.7
Use sprinkler to avoid dusting	2	2.5
Add heat to avoid caking of litter	26	32.1
Till the litter	15	18.5
Move water lines	10	12.4
Add litter treatment products	2	2.5
Adjust ventilation	7	8.6
*Adding extra bedding during production (n = 82)*		
No	9	11.0
Yes	73	89.0
Around water lines	48	58.5
Between water and feed lines	36	43.9
Around perimeter	1	1.2
Entire barn	14	17.1
*Adding products to the feed/water to reduce wetness (n = 80)*		
Yes	13	16.3
No	67	83.8
*Previous flock reared on the litter (n = 83)*		
Yes	13	15.7
No	70	84.3

**Table 4:** Frequency of feed and water management practices for 83 turkey flocks placed between October 2018 and December 2019 in Canada. The total number of responses is given for each individual question.

	Responses	
	n	%
*Feed structure (n = 79)*		
Mashed feed	12	15.2
Pelleted feed	59	74.7
Crumbs	4	5.1
Combination of crumbs/pellets	4	5.1
*Feed source (n = 82)*		
Home-milled	12	14.6
Purchased	70	85.4
*No. of times diet has been changed (n = 80)*		
No changes	5	6.3
1–2x	7	8.8
3–4x	28	35.0
5–6x	31	38.8
7–8x	8	10.0
More than 8x	1	1.3
*Feed supplements provided (n = 76)*		
No	40	52.6
Yes	36	47.4
Insoluble grit	24	32.5
Insoluble fiber	8	10.4
Vitamins/minerals	16	20.8
Other	1	1.3
*Animal by-products in feed (n = 62)*		
Yes	21	33.9
No	41	66.1
*Watering system (n = 82)*		
Closed watering system (i.e., nipple drinker with/without cup)	57	69.4
Open watering system (i.e., bell drinkers or troughs)	25	30.5
*Water supplements provided (n = 82)*		
No	37	45.1
Yes	45	54.9
Acids	33	40.2
Vitamins/minerals	22	26.8
*Flushing of water lines before placement (n = 83)*		
Yes	80	96.4
No	3	3.6
*Water sanitizing products added (n = 82)*		
No	12	14.6
Yes	70	85.4

**Table 5:** Frequency of environmental control practices for 83 turkey flocks placed between October 2018 and December 2019 in Canada. The total number of responses is given for each individual question.

	Responses	
	n	%
*Type of lighting ^1^ (n = 82)*		
Incandescent	9	11.1
Fluorescent	10	12.4
LED	63	77.8
Natural	3	3.7
*Light intensity (n = 64)*		
Less than 10 lux	19	29.7
10–20 lux	23	35.9
21–30 lux	14	21.9
More than 30 lux	8	12.5
*Dark period provided (n = 80)*		
Yes	73	91.3
No	7	8.8
*Intermittent light program used (n = 77)*		
Yes	14	18.2
No	63	81.8
*Housing system (n = 81)*		
Natural (open-sided barn with curtains)	30	37.0
Power (controlled environment housing)	51	63.0
*Ventilation (n = 82)*		
All natural	9	11.0
Mechanical (fans)	51	62.2
Mixed	22	26.8
*Type of ventilation (n = 69)*		
No mechanical ventilation	9	13.0
Cross flow ventilation	47	68.1
Tunnel flow ventilation	6	8.7
Cross and tunnel flow hybrid	7	10.1

^1^ Farmers could select multiple options.

**Table 6:** Characteristics of 83 turkey flocks placed between October 2018 and December 2019 in Canada. The total number of responses is given for each individual question.

	Responses	
	n	%
*Sex (n = 83)*		
Hens	53	63.9
Toms	30	36.1
*Physical alterations (n = 79)*		
Beak trimming	78	98.7
Toe treatment	45	57.0
Dew claw removal	17	21.5
Snood removal	22	27.9
*Brooding barn (n = 80)*		
Brood to finish growing system (current barn)	25	31.3
Brood and move growing system (brooding barn)	55	68.8
*Issues observed upon move into the grow-out barn ^1^ (n = 54)*		
No	21	38.9
Yes	33	61.1
Variation in body size	21	38.9
Leg injuries	6	11.1
Leg deformities	17	31.5
Wing injuries	5	9.3
Blood on plumage	3	5.7
Feather damage	3	5.6
*Strain*		
Hybrid Converter	59	78.7
Aviagen Nicholas Select	21	28.0
Orlopp Bronze	1	1.3

^1^ Only answered by farmers who had moved birds to the grow-out barn.

**Table 7:** Characteristics of farm biosecurity management of 83 turkey flocks placed between October 2018 and November 2019 in Canada. The total number of responses is given for each individual question.

	Responses	
	n	%
*Barn preparation for arrival (n = 81)*		
Dry cleaning	54	66.7
Wet cleaning	59	72.8
Disinfection	58	71.6
Downtime period	53	65.4
Fumigation	18	22.2
Heat treatment	8	9.9
*Biosecurity practices (n = 82)*		
Vermin control	70	85.4
Inspect age-wise	56	68.3
Downtime period after farm contact	19	23.2
Washing of boots/boot dips	68	82.9
Single-use boot covers	27	32.9
Handwashing before entering	36	43.9
Specific clothes for each barn	28	34.2
Shower in/out	6	7.3
*Age class (n = 82)*		
Single age farm	24	29.3
Multi-age farm	58	70.7

**Table 8:** Characteristic flock health management of 83 turkey flocks placed between October 2018 and December 2019 in Canada. The total number of responses is given for each individual question.

	Responses	
	n	%
*Flock certification (n = 78)*		
None (conventional)	22	28.2
Raised Without Antibiotics/Antibiotic Free	28	35.9
Responsible Use of Antibiotics	27	34.6
Organic	1	1.3
*Vaccination at hatch/farm (n = 79)*		
Yes	51	64.6
No	28	35.4
*Existing relationship with the veterinarian (n = 83)*		
Yes	76	91.6
No	7	8.4
*Medication administered (n = 78)*		
Yes	31	39.7
No	47	60.3
*Disease in flock (n = 71)*		
Yes	21	29.6
No	50	70.4
*Hospital/recuperation pens (n = 82)*		
Yes	30	36.6
No	52	63.4
*Euthanasia certified by veterinarian/professional (n = 82)*		
Yes	73	89.0
No	9	11.0
*No. of bird inspections per day (n = 83)*		
1x	2	2.4
2x	47	56.6
3x	24	28.9
4x	3	3.6
More than 4x	7	8.4
*No. of workers (n = 82)*		
1 worker	37	45.1
2 workers	38	46.3
3 workers	7	8.5
*Average time spent on inspection per day (n = 83)*		
Less than 15 min	13	15.7
15–30 min	54	65.1
30–45 min	12	14.5
45–60 min	2	2.4
More than 60 min	2	2.4
